# Mining for genotype-phenotype relations in Saccharomyces using partial least squares

**DOI:** 10.1186/1471-2105-12-318

**Published:** 2011-08-03

**Authors:** Tahir Mehmood, Harald Martens, Solve Sæbø, Jonas Warringer, Lars Snipen

**Affiliations:** 1 Biostatistics, Department of Chemistry, Biotechnology and Food Sciences, Norwegian University of Life Sciences, Norway; 2 Centre of Integrative Genetics (CIGENE), Norway; 3 Department of Mathematical Sciences and Technology, Norwegian University of Life Sciences, Norway; 4 Department of Cell and Molecular Biology, University of Gothenburg, Sweden

## Abstract

**Background:**

Multivariate approaches are important due to their versatility and applications in many fields as it provides decisive advantages over univariate analysis in many ways. Genome wide association studies are rapidly emerging, but approaches in hand pay less attention to multivariate relation between genotype and phenotype. We introduce a methodology based on a BLAST approach for extracting information from genomic sequences and Soft- Thresholding Partial Least Squares (ST-PLS) for mapping genotype-phenotype relations.

**Results:**

Applying this methodology to an extensive data set for the model yeast *Saccharomyces cerevisiae*, we found that the relationship between genotype-phenotype involves surprisingly few genes in the sense that an overwhelmingly large fraction of the phenotypic variation can be explained by variation in less than 1% of the full gene reference set containing 5791 genes. These phenotype influencing genes were evolving 20% faster than non-influential genes and were unevenly distributed over cellular functions, with strong enrichments in functions such as cellular respiration and transposition. These genes were also enriched with known paralogs, stop codon variations and copy number variations, suggesting that such molecular adjustments have had a disproportionate influence on *Saccharomyces *yeasts recent adaptation to environmental changes in its ecological niche.

**Conclusions:**

BLAST and PLS based multivariate approach derived results that adhere to the known yeast phylogeny and gene ontology and thus verify that the methodology extracts a set of fast evolving genes that capture the phylogeny of the yeast strains. The approach is worth pursuing, and future investigations should be made to improve the computations of genotype signals as well as variable selection procedure within the PLS framework.

## Background

The current growth in genomic data demands new or improved methods for exploring the genotype-phenotype landscape. Due to the complexity of the cellular interaction networks, polymorphisms in individual genes often have only a weak association with the variation in common traits. However, as phenotypes result from the functional interactions between the products of different genes, the association between genotype and phenotype may be captured from co-occurrence of multiple genes and multiple phenotypes across a wide range of individuals. Recent development in statistical methods and phylogenetics are addressing these issues [1,2].

The yeast *Saccharomyces cerevisiae *has a long history as a key model organism in molecular and cellular biology and is rapidly emerging as a prime experimental system also for achieving an organism-wide bridging of the gap between genotype and phenotype [3-9]. These studies are based on linkage analysis [3], population genetic analysis [4], correlation analysis [6,9], gene knockout sensitivity measure [8], and gene knockout genetic interaction networks [7], mutual information to evaluate the biconditional relation [2] as well as a probabilistic model [5] for mapping genotypes on phenotypes. However, these approaches are intrinsically limited by the fact that they pay little attention to the multivariate relation between genotypes and phenotypes, i.e. they do not simultaneously consider the impact of more than one gene on more than one phenotype.

The use of multivariate approaches in genome-wide association analysis may be expected to pro-vide decisive advantages over univariate analysis in many ways. Firstly, a fundamental lesson learned from genome-wide association studies is that most phenotypes, including many common diseases, seem to be complex. Not only are they highly polygenic, but, it is typically found that only a fraction of the total phenotype variation is explained by summing up the significant contributions of individual genes. This is partially believed to reflect the importance of non-additive genetic interactions between genes, which cannot be captured by univariate approaches [10]. Secondly, assuming that the correlation between phenotypes is partly due to the shared effect of a suite of genes, multivariate analysis making simultaneous use all of the available phenotypes is intrinsically more powerful than several repeated univariate analysis that consider each phenotype separately [11]. Thirdly, the correlation among phenotypes is in itself of key scientific interest, whether it is due to pleiotropic (i.e., multifunctional) genes or shared genes with tightly linked functions [12]. For example, orphan drugs may be assigned mechanisms of action on the basis of close correlation to drugs with known targets using a guilt-by-association principle [13,14]. And fourthly, by considering aggregate effects, multivariate analysis can increase the sensitivity to identifying important genetic effects and detect contributions of genetic variants that have too small effect to be detected by univariate analysis [15]. Hence, multivariate analysis has the potential to provide superior statistical power, increased interpretability of results and a deeper functional understanding of the gene-phenotype landscape; consequently, the development of efficient multivariate approaches in genetics should be of high priority.

Although multivariate analysis has been introduced in genome-wide association analysis [16,17] it has not been fully established. There are several multivariate methods that are extensively applied in other scientific field that could potentially be used to explore the genotype-phenotype landscape (e.g. [18,19]). In essence, multivariate methods consider the covariance structure of genotypes and phenotypes and identify combinations of influential genotypes that map to combinations of phenotypes. The initial step of any multivariate based dissection of genotype-phenotype relations is the computation of numerical features from the sequence data. Some approaches are based on word frequencies or their modifications [20-22]. SNPs are also considered as gene markers as their lower polymorphism is offset by their abundance and ease of genotyping and their low mutation rates make them especially suitable for linkage mapping, i.e. the co-inheritance of genotypes and phenotypes in successive generations [23]. In this paper, we demonstrate the feasibility and power of an alternative approach: computing features from genome sequences by considering the degree of similarity to a set of reference sequences. Each genome is compared to this reference genome by pair-wise alignment, and for each reference sequence we get a normalized score, indicating to what extent it is found in the respective genome. A similar approach has also been employed in evolutionary studies based on whole-genome data, more specifically, in the construction of gene-content trees [24,25].

This feature computation provides us with a data set having a large number of reference sequences *p *in comparison to the number of genomes *n*; for example, for the unusually gene-dense baker's yeast we have in this study used 5791 reference sequences (genes) and 36 genomes. This 'large *p *small *n*' situation makes it difficult to relate a certain phenotypic response to a reasonably sized subset of reference sequences, as there is not enough information in the data to find unique estimates for regression coefficients that best fit the data in the ordinary least squares sense. Feature selection based on some pre-association analysis may be needed in order to eliminate unrelated features (noisy features) and include only a modest number of presumably more relevant genotypes in the analysis [26]. It is also important to group the phenotypes by their common characteristics over genomes; for example, this may allow the assignment of mechanisms of action of orphan drugs on the basis of clustering with drugs that have known cellular targets [13,14]. Multivariate tools, like Partial Least Square (PLS) regression, are widely used in chemometrics to address the problem of making good predictions in the 'large *p *small *n*' situation [27]. In later years there has also been an increase in applications using PLS in bioinformatics research (e.g. [28-30]). In principle, the PLS algorithm will try to identify a relevant subspace in the genotype space which explains the maximum variance in the phenotype space. Based on the latent components spanning this subspace a bilinear regression model is constructed for the prediction of phenotypes. Unfortunately, PLS in its original form has no implementation for feature selection, more specifically, no selection procedure for phylogenetic genes that best explain the genotype-phenotype relation. One possible way is to use PLS in combination with jackknife testing [31], which is a resampling method for performing statistical inference about the regression coefficients. However, it is not entirely clear that it will indeed select a reasonable set of genotypes when the reference sequence versus number of genomes gets as large as is the case here. In [18] a soft- thresholding step in the PLS algorithm is suggested, based on ideas from the nearest shrunken centroids method [32]. This ST-PLS algorithm per-forms a simultaneous model fitting and feature selection. In this paper, we exemplify the applicability of ST-PLS when employed in the unification of the high dimensional genotype space and the phenotype space in order to unravel associations for subsequent in-depth studies.

## Methods

### Approach

#### Data

Genome sequences for 36 *Saccharomyces cerevisiae *strains were obtained from the Saccharomyces Genome Resequencing Project (SGRP) and are publicly available at Sanger http://www.sanger.ac.uk/Teams/Team118/sgrp/. Genomic sequences representing 1.3-12-fold coverage correspond to a nuclear genome of 16 chromosomes and a mitochondrial DNA. The description of these genomes is given in Additional file 1, Table S1. The universal yeast reference strain S288C, the first eukaryotic genome to be sequenced [33] and the reference genome for several whole genome approaches [34], was used as a reference genome also here. In principle, any sequence feature of S288C, including rRNA, tRNA, snRNA, transposons and promoter regions could be considered as reference sequence elements; however to reduce the search space to be tested in this proof of principle study we here restricted the analysis to protein coding genes which are directly related to phenotypes. In total 6850 protein-coding sequences were downloaded from the Saccharomyces Genome Database (SGD) http://www.yeastgenome.org/ and used as reference sequences in this study. Dubious genes which were not conserved across closely related genomes [35] and all putative ORFs that were not stringently annotated as genes were excluded, leaving 6067 genes. As explained below, some of these genes did not give a good spread in evolutionary distances over the 36 genomes, and were discarded as un-informative in a final step, resulting in a set of 5791 genes in this study.

The phenotype data were obtained by micro-cultivation of yeast populations during exposure to 10 different treatments, representing a wide diversity of natural and artificially imposed environment variations [36]. Sigmoid growth curves were parameterized as described [37] into the two fundamental reproductive measures the reproductive rate (doubling time, Rate) and the reproductive efficiency (gain in population density given the available resources, Efficiency). The Rate was defined by the slope in the exponential phase converted into population doubling time and the Efficiency (optical density units) was defined as the total change in density. Detailed descriptions of these growth variables can be found in [38]. We selected ten environments that correspond to known variations in yeast ecological niches and evaluated our approach on the basis of growth rate and growth efficiency data obtained from strains growing in these environments. Any missing phenotype values were imputed using the K-nearest neighbor method, in terms of overall phenotype pattern. In total, *v *= 20 distinct phenotype measures for *n *= 36 genomes, were retained for downstream analysis.

#### Genotype-phenotype relations

Data for each phenotype was assembled into a column vector ***y ***of length *n *= 36. Each genome sequence element, i.e. protein coding gene element, was converted into a vector of numeric features by sequence alignment to the corresponding reference sequence element, see Methods section. This was assembled into an *n *× *p *matrix ***X ***with *n *= 36 rows and *p *= 5791 columns, one column for each reference sequence element. To mine for relations between phenotypes and genotypes, we implemented a Partial Least Squares (PLS) approach [27]. There are many variants of the PLS modeling paradigm [18,19,39-41]; here we employed the Soft Threshold PLS [18] which is specifically designed for multivariate feature selection such as phylogenetic genes that are called for in defining genotype-phenotype associations. In essence, the concept means that we are looking for combinations of columns of ***X ***capable of explaining the variations in each ***y***,(see the Methods section for details).

#### Integrating external genotype features

The S288C genome is exceptionally richly and coherently annotated on a functional level, reflected in that Gene Ontology (GO) annotations [42] exist for more than 80% of its protein coding genes. This abundance of structured functional information allowed unbiased evaluation of the derived genotype-phenotype associations. Gene Ontologies (GO) were obtained from the Yeast GO Slim Mapper http://www.yeastgenome.org/cgi-bin/GO/goSlimMapper.pl in the form of three distinct functional annotation sets: the major biological processes in which genes are involved (45 categories); their molecular/biochemical activities (25 categories) and the cellular components in which the corresponding protein has been found (25 categories). A single gene may be mapped to multiple GO terms. Interpretation of genotype-phenotype associations was also performed taking gene essentiality/non-essentiality http://www.yeastgenome.org in the S288C background into ac-count as well as data on whether a gene is present in S288C as a singleton or as a duplicated gene (a paralog); the latter was defined as having a blastp hit among other S288C genes with *E <*10^-10 ^over at least 50% of its length. Finally, we also mined the genotype-phenotype associations taking the molecular basis of the genotype polymorphisms in *S. cerevisiae *into account. Genes with polymorphisms presumed to be strongly associated to phenotypes, stop codon mutations, frame shifts and copy number variations, as identified in the *S. cerevisiae *lineages [9] were analyzed as distinct classes.

### Algorithm

#### Computing genotype scores

First, all reference sequences (translated nucleotide) were aligned against themselves using score 1 for match. In this way the maximum alignment score *S*(*r_j _*; *r_j _*) was obtained for each reference sequence *r_j _*representing some coding gene of the S288C genome. This score corresponds to the length of sequence *r_j_*. Then each *S. cerevisiae *genome was BLASTed against this reference set, using tblastx http://blast.ncbi.nlm.nih.gov/Blast.cgi?CMD=Web&PAGE_TYPE=BlastHome. Hence for each genome sequence *g_i _*a maximum bit-score, *S*(*g_i_*; *r_j _*), was obtained indicating to what extent sequence *r_j _*was found in the respective genome. Since this score depends heavily on the length of the aligned sequences, we used the normalized score

where the lower bound 1/20 is used for computational reasons when using the Jukes-Cantor transformation below. Reference sequences where all the normalized scores were below 0.5 were discarded from the downstream analysis. The reasoning be-hind this is that sequences with no clear similarities in any genome are probably introducing more noise than information. This filtering produced a final set of 5791 reference sequences.

Finally we have used Jukes-Cantor evolutionary model [43] to extract the numerical feature in ***X***, i.e.

Thus, the genotype variables are approximate evolutionary distances from the reference genome. The minimum distance is *X*_*i*,*j *_= 0, indicating that the reference sequence *r_j _*is found with 100% identity in genome *g_i_*. These features were assembled into the genotype matrix ***X ***= {*X*_*i*,*j*_} having one row for each of the 36 genomes and one column for each of the 5791 reference sequences.

#### ST-PLS supervised learning

The association between a phenotype vector ***y ***and the genotypes ***X ***was assumed to be explained by the linear model E(***y***) = ***Xβ ***where ***β ***are the *p *× 1 vector of regression coefficients. The main purpose of the study was to find the subset of genotypes best explaining the variations in each phenotype. From a modeling perspective, ordinary least square fitting was no option since the number of samples (n = 36) was much smaller than the number of features (p = 5791). PLS resolves this by searching for a set of components, "latent vectors", that performs a simultaneous decomposition of ***X ***and ***y ***with the constraint that these components explain as much as possible of the covariance between ***X ***and ***y***.

Prior to all model fitting, all variables in ***y ***and ***X ***were centered and standardized by subtracting the column mean and dividing by the standard deviation.

The PLS estimate of the regression coefficients for the above given model based on *k *components can be achieved by(1)

where  is the *p *× *k *matrix of ***X***-loadings,  is the *k *vector of ***y***-loadings and  is the *p *× *k *matrix of loading weights as defined in [27]. Selection of variables based on the magnitude of PLS loading weights (the columns of ) is an accepted approach, and [18] suggested a soft-thresholding step in the PLS algorithm based on ideas from the nearest shrunken centroids method [32]. At each step of the sequential PLS algorithm the weights were modified as

i) Scaling:

***w***_*k *_← ***w***_*k*_/max_*j *_|*w*_*k*,*j *_|, for ***j ***= 1, ..., *p*

ii) Soft-thresholding:

*w*_*k*,*j *_← sign(*w*_*k*,*j*_)(|*w*_*k*,*j *_| -*δ*)_+_, for *j *= 1, ..., *p*.

Here (...)_+ _means max(0, ...)

iii) Orthogonalization:

iv) Normalizing:

***w***_*k *_← ***w***_*k*_/||***w***_*k*_||

The shrinkage *δ *∈ [0, 1) decides the degree of thresholding, i.e. a larger *δ *gives a smaller selected set of genes in the genotype-phenotype mapping. Cross validation was used to find the optimal number of components and shrinkage level *δ*, and in this study a random leave 3-out cross-validation scheme was chosen. For each left-out segment ST-PLS models with 1 to 10 components in combination with shrinkage levels (0.70, 0.73, 0.76, 0.79, 0.82, 0.85, 0.88, 0.91, 0.94, 0.97) were fitted and the left out samples were predicted. After cycling through all sample segments, the root mean square error (CVRMSE) of prediction was computed for each component/threshold combination in search for the best model. The minimum CVRMSE is itself a stochastic variable, and in the search for the optimal number of components and shrinkage level, a slack in CVRMSE corresponding to two standard errors was allowed. In this way a reduction in model complexity (number of components) and/or an increased shrinkage level away from the apparent optimum was allowed as long as the CVRMSE was below the minimum plus slack. This allowed us to select a reasonable number of associated genes for all phenotypes.

For evaluation of model performance an index of agreement d-statistics was used [44], which reflects the degree to which the observed response is accurately estimated by the predicted response. It varies from 0 (complete disagreement between predicted and observed responses) to 1 (perfect agreement).

## Results

### Genotype-phenotype modeling

In order to study the relationship between genetic and phenotypic variation in yeast an ST-PLS model was fitted for each of the 20 phenotype responses, as described in the Method section. A genotype predictor matrix was derived by blasting the genes of each genome to a *S. cerevisiae *reference genome and the best hit scores were used as numerical inputs to this genotype matrix. This provides resolution in terms of each individual polymorphism, which vastly reduces the complexity and provides sufficient power to statistically link variation in genes to variation in phenotypes.

**Figure 1:**
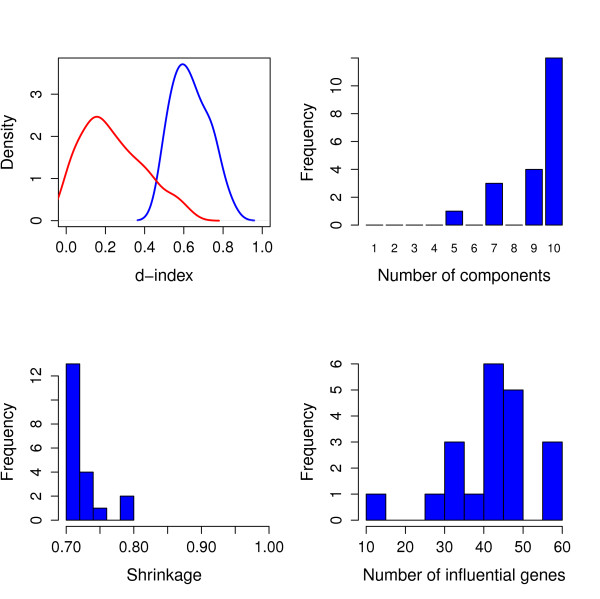
**Over all distribution of model parameters and performance**. Results obtained from the 20 ST-PLS model fits. The upper left panel shows the distribution of the d-index, which is a measure for a models explanatory power (range 0-1) obtained from the cross validation by using the finally selected shrinkage level and a number of components. The blue curve indicate its distribution for our 20 phenotypes, and the red curve is the distribution of this measure if the ***X ***matrix is replaced by a random shuffling of its rows, i.e. a 'null'-distribution. The upper right panel shows the distribution of the number of components selected (range 1-10), the lower left panel similar for shrinkage level (range 0.7-0.96) and the lower right panel similar for the number of associated genes.

In Figure 1 some results for the fitting of all 20 phenotypes are displayed. For each fitted ST-PLS model the performance statistics d-index [44] was computed, and in the upper left panel we can see how this distributes over the 20 phenotypes (blue curve). For comparison we have also included a 'nulldistribution' of this statistic found by randomized reshuffling of the data (red curve). This demonstrates that we in the vast majority of cases are able to find stable explanation of the phenotypes by combining genotype information.

In the upper right panel we observe that the genotype-phenotype mapping is in most cases found with more than six components. This indicates that several genes are associated with a certain phenotype, and that these genes contribute with different information such that the phenotype can only be explained by combining six or more directions in genotype space. Further, in Additional file 2, Figure S1, the upper panel indicates that the complexity of the model increases with decreased shrinkage level.

In the lower left panel we find that the optimal shrinkage level is in most cases moderate, and never above 0.8. This is partly due to our constraint that we require at least 25 genes selected, but it also tells us that the associated genes 'stand out' and can be identified without using extremely high shrinkage levels.

The lower right panel is a histogram over the number of associated genes found for each of the 20 phenotypes. In the Additional file 2, Figure S1 the lower and middle panels indicate that the number of influential genes increases with model complexity and decreases with shrinkage level.

### Distribution of associations

A key assumption of genetics is that traits are controlled by subsets of genes that are largely distinct but that overlaps between traits that share functional elements. Such genes that control multiple traits are referred to as pleiotropic [45]. The phenotypes included here all represent different environ-mental stresses and as such, reflects the highly generalized environmental stress response [46,47]. Given that the method extracts relevant biological information, we expected substantially higher pleiotropy than by any random selection of genes. This was indeed the case. Considering all phenotypes, the gene influence on trait variation was highly unevenly distributed. Variation in certain genes tended to define many phenotypes. 14 to 60 genes explained 50% to 88%of the total phenotypic variation, whereas some 5699 genes did not noticeably influence the overall phenotype variation. This highly skewed distribution of gene influences deviated significantly from the results of a simulation study using random genotypes, where approximately 200 times as many genes were found to 'affect' phenotypes. The random genotypes were simulated by random permutations of the rows of ***X***.

**Figure 2:**
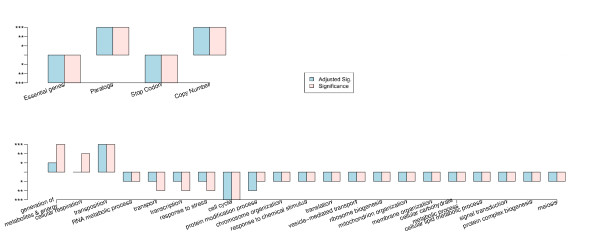
**Overall enrichments**. Certain types of variations that are over represented (positive bars) and under represented (negative) among the overall influential genes for all phenotypes. The upper panel includes the variations like essential genes, genes with known paralogs, genes with known frame shift variation, genes with known stop codon variation and genes with known copy number variations in yeast. The lower panel includes enriched Gene Ontology process terms. On the y-axis significance at 10% is marked with *, 5% is marked with ** and 1% is marked with ***. Variations are also marked with significance based on adjusted p-values (False Discovery Rate adjusted).

A central assumption of genetics is that different types of genetic variations differs in their impact on traits depending on how they affect the quantity and quality of the final product produced, in most cases the proteins. For example, genetic variation resulting in premature termination of translation, e.g. premature stop codons, is expected to have a disproportionately large impact on trait variation as these variations directly affect the quality of the translated protein. Similarly, recent and older gene duplications, reflected in gene copy number variations and paralogous gene pairs respectively, are widely assumed to provide adaptive trait variation as it both increases protein and allows for evolution of novel functions while maintaining the original function. Hence, we expected an influence of genes known to harbor such variation on the studied traits. This was indeed the case, as seen in Table 1. Both genes with gene copy number variations (*p <*0.01) and gene paralogs (*p <*0.01) were over-represented as affecting the studied traits, see Figure 2.

**Table 1:** Enriched variations

Label	Phenotype	*N*	Ess. genes	Paralog	Frame shifts	Stop codon	Copy no.
Mel_R	Melibiose 2% Rate	33	0	2.06^*^	0.25	1.23	4.41^*^
Mel_E	Melibiose 2% Efficiency	40	0	0.78	0.20	1.37	3.59
Cup_R	Cupper chloride 0.375 mM Rate	60	0.16	2.56^***•••^	0.15	1.63	6.42^***•••^
Cup_E	Cupper chloride 0.375 mM Efficiency	14	0	2.19	0.11	3.36^*^	11.42^**•^
NaC1_R	NaCl 0.85 M Rate	58	0.16	2.91^***•••^	0.05	1.16	8.25^***•••^
NaC2_R	NaCl 1.25 M Rate	47	0.31	1.12	0.13	1.46	0
NaC1_E	NaCl 0.85 M Efficiency	47	0.01	2.34***^••^	0.13	1.46	12.70^***•••^
NaC2_E	NaCl 1.25 M Efficiency	43	0.11	1.25	0.14	0.92	3.33
Mal_R	Maltose 2% Rate	59	0.51	2.05^**••^	0.19	1.39	11.50^***•••^
Mal_E	Maltose 2% Efficiency	45	0.32	1.37	0.21	0.87	13.37^***•••^
Gal_R	Galactose 2% Rate	30	0	1.67	0	0.88	22.11^***•••^
Gal_E	Galactose 2% Efficiency	49	0.19	2.67^***•••^	0.27	1.40	22.11^***•••^
Hea1_R	Heat 37°C Rate	33	0	2.06^*•^	0.09	2.20^*^	9.65^***•••^
Hea2_R	Heat 40°C Rate	44	0	2.06^**•^	0.07	1.23	13.73^***•••^
Hea1_E	Heat 37°C Efficiency	44	0.11	1.21	0.26	1.58	1.568
Hea2_E	Heat 40°C Efficiency	49	0.40	1.78^*^	0.12	1.72	9.99^***•••^
Sod1_R	Sodium arsenite oxide 3.5 mM Rate	48	0	5.58^***•••^	0.13	2.11^*•^	12.38^***•••^
Sod2_R	Sodium arsenite oxide 5 mM Rate	33	0.29	3.15^***••^	0.09	2.20^*^	2.11
Sod1_E	Sodium arsenite oxide 3.5 mM Efficiency	44	0.22	1.83^*^	0.18	1.95	13.73^***•••^
Sod2_E	Sodium arsenite oxide 5 mM Efficiency	43	0	3.62^***•••^	0.14	2.40^**•^	3.33

Certain types of variations that are over-represented among the *N *influential genes for all phenotypes. The statistics are odds-ratios indicating potential enrichment of certain gene categories among the influential genes. The categories are: Essential genes, genes with known paralogs, genes with known frame shift variation, genes with known stop codon variation and genes with known copy number variations in yeast. Significance at 10% is marked with ^*^, 5% is marked with ^** ^and 1% is marked with ^***^. The corresponding significance based on adjusted p-values controlling the false discovery rate (q-values) are marked with _•_, _•• _and _•••_, respectively.

Based on computations of the frequency of non-synonymous versus synonymous variations that have emerged since the split between S. cerevisiae and its closest relative Saccharomyces paradoxus [48], we found the here identified influential genes to have been evolving 20% faster than non-influential genes (ratios 0.100 vs 0.078, t-test, *p <*0.10). This indicates that these genes, as a group, have been subjected to either somewhat stronger positive selection, or somewhat relaxed negative selection during the recent yeast history. This was expected, genes affecting traits, should do so either through adaptive variation or through genetic variation that is neutral in the local environment, implying an elevated rate of evolution. However, the difference is limited and the bias, approximately 10:1, against non-synonymous mutations in these genes has nevertheless been strong. The observation that genes associated to phenotypic variation correspond to genes with an elevated rate of evolution, suggests that they affect a a nonrandom set of cellular functions. In essence, we expect this gene set to be enriched for genes that regulate the relation between the organism and its environment. From Table 2 we can see that the various lists of influential genes frequently support enrichments obtained from the Fisher exact test in categories such as generation of precursor metabolites and energy (*p <*0.1), cellular respiration (*p <*0.1) and transposition (*p <*0.01).

**Table 2:** Enriched Gene Ontology

Label	GO terms
Mel_R	transposition^**^
Mel_E	generation of precursor metabolites and energy^***•^; cellular respiration^***•^
Cup_R	cellular respiration^*^; transposition^***•••^
Cup_E	generation of precursor metabolites and energy^***••^; heterocycle metabolic process^*^; cellular respiration^**^; transposition^**^
NaC1_R	cellular respiration^*^; transposition^***•••^
NaC2_R	generation of precursor metabolites and energy^**^; cellular respiration^**^; transposition^***•••^
NaC1_E	generation of precursor metabolites and energy^**^; transposition^***••^
NaC2_E	generation of precursor metabolites and energy^**^; transposition^***•••^
Mal_R	generation of precursor metabolites and energy^**^; cellular respiration^*^; transposition^***•^
Mal_E	generation of precursor metabolites and energy^*^; transposition^***•^
Gal_R	generation of precursor metabolites and energy^*^; cellular respiration^*^
Gal_E	generation of precursor metabolites and energy^***•^; cellular respiration^***•^
Hea1_R	generation of precursor metabolites and energy^***•^; heterocycle metabolic process^*^; vesicle organization^**^
Hea2_R	generation of precursor metabolites and energy^**^; transposition^***•^
Hea1_E	generation of precursor metabolites and energy^**^; transposition^***•••^; vesicle organization^**•^
Hea2_E	generation of precursor metabolites and energy^***•^; heterocycle metabolic process^*^; cellular respiration^**^; transposition^**•••^
Sod1_R	transposition^***•••^
Sod2_R	generation of precursor metabolites and energy^*^; transposition^***•••^
Sod1_E	DNA metabolic process^*^; generation of precursor metabolites and energy^**^; cellular respiration^**^; transposition^***••^
Sod2_E	transposition^***•••^

Enriched Gene Ontology process terms are listed. Significance at 10% is marked with ^*^, 5% is marked with ^** ^and 1% is marked with ^***^. The corresponding significance based on adjusted p-values controlling the false discovery rate (q-values) are marked with _•_, _•• _and _•••_, respectively.

**Figure 3:**
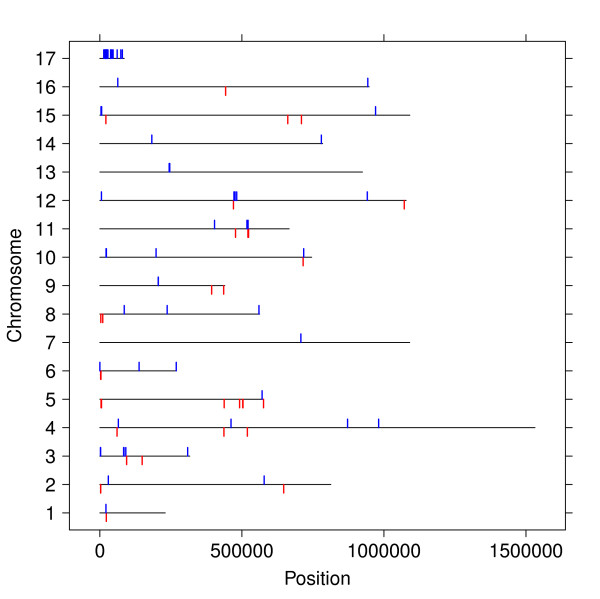
**Distribution of genes on chromosomal positions**. The distribution of all genes related to at least one phenotype over the 16 chromosomes of *S. cerevisiae *strain S288C. Blue tags indicate a gene on the positive strand and red tags on the negative strand.

In contrast, the genes we found associated to phenotypes were fairly well scattered across locations along all 16 chromosomes, as indicated in Figure 3, also with regards to subtelomeric regions, which frequently show rapid evolution.

### Dissecting multivariate gene-phenotype associations

A fundamental biological question is what types of genes and genetic variations that define the main structures of variation in distinct traits, within a species. To exemplify the applicability and power of the ST-PLS procedure we performed an in depth analysis of two environmental traits, that are highly variable between strains [9] and that are believed to have a complex structure, NaCL1_E (NaCl 0.85 M Efficiency) and Hea1_R (Heat 37°C Rate).

**Figure 4:**
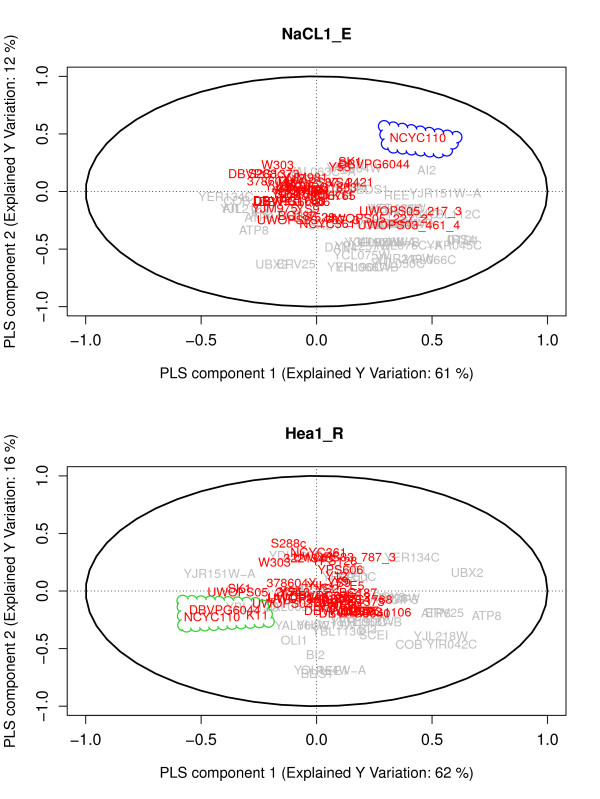
**Biplots for NaCL1_E and Hea1_R**. The biplot for NaCL1_E (NaCl 0.85 M Efficiency) in upper panel and for Hea1 R (Heat 37°C Rate) in lower panel is presented. Genes are labeled by their names in gray color and strains are indicated by red color. For the model NaCL1_E most variant strain NCYC110 is identified and is marked by the blue cloud. For Hea1_R two most variant strains NCYC110 and DBVPG6044 are identified with their related genes in a green cloud.

The yeast response to high concentrations of sodium, which imposes both ion and osmotic strain on the cell, is one of the best understood responses to a variation in the external milieau [49]. We found that the variation in cellular growth efficiency during exposure to 0.85 M NaCl in the *S. cerevisiae *strains was largely controlled by 47 associated genes, i.e. 47 genes are frequently associated with the phenotype. The optimal number of components for the ST-PLS model was 10, indicating a complex relation between genotype and this phenotype. Among the 47 influencing genes there was an enrichment of genes that have paralogs, i.e. genes that have been at least duplicated in ancestral times in the reference strain S288C, and of genes that vary in copy number within baker's yeast, i.e. genes that have undergone very recent duplications in some strains (Table 1). They were also enriched for generation of precursor metabolites and energy and transposable elements (Table 2). In contrast, we found no significant overlap to loss of genes which is known to lead to defects in the salt response in S288C [50]. This is partially explained by the high degree of conservation in many of the genes most important for salt tolerance in S288C; for example, the HOG1 gene product which controls, expression of salt responsive genes in S288C was essentially invariable. Figure 4 upper panel shows the correlation biplot for NaCl 0.85 M over the first two PLS-components. The correlation biplot shows for each gene their contribution to the two dimensions or underlying phenomena (loadings), and for each strain their relative position in this two-dimensional space (scores). This identifies the most variant strain, NCYC110 of the West African population, and genes specifically related with variations in this strain. These genes were enriched for copy number variations, among them the known sodium exporters ENA1, 2 and 5 genes, which are present in three copies in yeast populations with high salt performance but in only one, genetically deviating copy, referred to as ENA6, in the West African population (Warringer et al, manuscript in preparation).

The yeast responses to high temperatures is less well understood than the salt response, but is nevertheless of high interest, as elevated tolerance to such variations is considered to be a primary feature of clinically relevant strains [51]. The growth rate variations in *S. cerevisiae *during exposure to heat 37°C, was largely defined by 33 genes, i.e. 33 genes are frequently associated with the phenotype in natural yeast stains. A 7 component model was optimal, indicating these 37 genes contribute with 7 different types of information in order to explain the phenotype. As for salt, this set of genes was enriched for genes that have paralogs in the reference strain S288C and for genes that vary in copy number between strains. In addition, it was enriched for genes known to harbor premature stop codons in some of the analyzed strains. The heat influencing gene set was also heavily enriched for genes involved in cellular processes such as generation of precursor metabolites and energy, heterocycle metabolic process and vesicle organization (Table 2). These processes are well known to be of importance for maintaining an optimal heat response, and are typically enriched in gene knockout screens for a defect heat tolerance [52]. Figure 4, lower panel, shows the correlation biplot for the phenotype Hea1_R over the first two PLS-components. Indicated are the two most variant strains, NCYC110 and DBVPG6044, the two identified strains of the West African population. Genes defining the low heat tolerance of NCYC110 were enriched for frame shift variations and genes related to heat tolerance variation in DB-VPG6044 were enriched for paralogs and copy number variations.

## Discussion

In this paper, we introduced a multivariate ST-PLS approach for mapping genotype-phenotype relations, using the well known reverse genetics model organism *S. cerevisiae *as a proof of principle. This approach requires the construction of a numerical genotype matrix which here was constructed by blasting a set of reference sequences against genomes. The usefulness of this approach of course depends on how well one can choose the reference set of sequences. We have in this case focused solely on protein coding sequences. One important reason for this is data reduction. Full genome sequences contain an overwhelming amount of potential information, and even with 36 strains the genotype subspace spanned in this data set is very limited in comparison. Also, by looking at coding genes one can focus on the part of the genomes believed to be most directly related to phenotype, anticipating that differences in the potential proteome between strains can explain some of their phenotypes. It is, however, possible to use exactly the same procedure as presented here for a bigger and more comprehensive set of reference sequences.

We have fitted one ST-PLS model for each phenotype, linking each phenotype to all genotypes. In Figure 1 we show some summary results for all 20 models. The results in the upper left panel of this figure ensure we have indeed found some stable relations between genotype and phenotype. This is a fundamental requirement for any further analysis. Every model will always come out with some 'best' relations, but such results cannot be trusted unless they are found to produce stable improvements in prediction performance.

From the upper right panel we see that in all cases, models with at least five components are needed to predict the phenotypes. This indicates rather complex relations between genotypes and phenotypes, in the sense that the associated genes contribute with different information, and several different combinations of genes are needed. Note that we distinguish between the number of associated genes and the number of directions/components when we talk about complexity. It is possible to have many associated genes, but still only a simple relation if all genes contribute with the same information, i.e. they are highly correlated. We also find that several associated genes are correlated since the number of genes is always much bigger than the number of components. It should also be noted that in our ST-PLS approach we are able to select all correlated genes as associated even if they contribute with the same information. Using for instance a stepwise selection procedure would not be capable of this, since the inclusion of one such gene will block the inclusion of another, correlated, gene.

The shrinkage level was in our analysis allowed to deviate from the optimum found by cross-validation, as explained in the Methods section. This allowed us to always select a reasonable number of associated genes (lower panels, Figure 1). In this proof-of-principle study we found it important to retain a comparable number of genes from each phenotype, in order to look for the enrichments of certain gene categories. It may of course be that we have included either too many or too few genes in some gene lists, but this can be sharpened in a more detailed study involving any specific phenotype.

In order to investigate whether the ST-PLS approach has picked up something essential, the overall trait-influencing genes common to at least 25% of the phenotypes were identified. These influential genes were enriched by genes with paralogs and genes with stop codons; this was entirely expected, given the assumed substantial phenotypic contribution of these genes. We also found a disproportionate influence of genes involved in mitochondrial respiration, which agrees with the influence of such genes on a wide variety of traits, including human diseases [53].

In contrast, the over-representation of genes involved in transposition, i.e. transposons, was some-what surprising. Few links between specific traits and transposons are known in baker's yeast, partially due to that the progenitor of baker's yeast show a selective loss of many classes of transposable elements [54] and partially due to the difficulties of studying such elements with reverse genetics. Nevertheless, the single class of transposons present in baker's yeast show dramatic variations in number and location between strains [9], and the phenotypic consequences in form of gene disruption are in some cases described, e.g. for the gene HAP1 which is rendered non-functional in S288C by transposon disruption, resulting poor performance in anaerobic and heme-depleted conditions [55]. In maize, where transposons have been most extensively studied, transposon mediated shuffling of genetic material is generally believed to be the main source of novel transcriptome [56].

We were also surprised to find that influential genes tended to be fairly uniformly distributed across all chromosomes, as seen in Figure 3. Adjacent genes in *S. cerevisiae *is known to show correlated gene expression, function annotation and gene knockout phenotypes [57,58], and the chromosomal ends are experiencing genetic churning [35], resulting in that essential genes and genes importance in multiple environments are preferentially kept away from chromosomal ends [59,60].

Overall, the influential genes were far from a random distribution but represented features with shared characteristics, supporting the validity, strength and robustness of the approach.

Figure 4 shows the dominating pattern of covariance between phenotypes and genotypes in two selected models. This pattern is dominated by a few *S. cerevisiae *strains. For growth efficiency in presence of high concentrations of salt (0.85 M NaCl), NCYC110 from the West African population had the strongest influence on the overall genotype-phenotype matrix. NCYC110 is known to have a poor salt tolerance [9]. Interestingly, NCYC110 genes linked to salt tolerance variation were enriched for copy number variations and these copy number variations influenced both PLS component directions.

For the growth rate at 37°C, not only NCYC110 but also its West African sister, DBVPG6044 had a strong overall influence. Both these strains have a severely reduced heat tolerance [9]. For NCYC110, genes with frameshifts were over represented among genes with a strong heat tolerance impact, as discovered earlier [61] whereas for DBVPG6044 genes with paralogs and copy number variations had disproportionate influence. The West African population is not genetically more distinct than any of the other known yeast populations, but on a phenotypic level it is highly unique, deviating in almost 30% of all traits (Warringer et al, manuscript submitted). Our study reinforces the view that these strains de-serve further attention from a perspective of revealing the structures underlying genotype-phenotype variations in natural populations.

## Conclusion

We have suggested a multivariate approach to the analysis of the genotype-phenotype mapping based on BLAST and PLS. We note that the derived results strictly adhere to the known yeast phylogeny and thus verify that the methodology extracts a set of fast evolving genes that capture the phylogeny of the yeast strains. We conclude that the approach is worth pursuing, and future investigations should be made to improve the computations of genotype signals as well as variable selection procedure within the PLS framework.

## Authors' contributions

The project and the coupling of multivariate methods to genotype-phenotype analyses, was formulated by JW, SS, HM and LS. TM, with some assistance from SS and LS, has done all programming and computations. TM, JW and LS drafted the manuscript. All authors have read and approved the final version.

## Supplementary Material

Additional file 1 **Table S1 - Saccharomyces strains**. Saccharomyces strains used in this study, along with their respective source location, source class and population & genomic structure are listed. Click here for file

Additional file 2 **Figure S1 - Mutual relation of shrinkage level, number of components and number of influential variables**. Results obtained from the 20 ST-PLS model fits presenting the mutual relation of shrinkage level, number of components and number of influential genes. Upper panel shows the scatterplot between the shrinkage level and a number component, indicating complexity of the model increases with decrease of shrinkage level. Middle panel shows the scatterplot between number of components and number of influential genes, indicating influential genes increases with the increase of model complexity. Lower panel shows the scatterplot between shrinkage level and number of influential genes, indicating influential genes decreases with the increase of shrinkage level. Click here for file
