# Supplementary figures and images for: Mining for genotype-phenotype relations in Saccharomyces using partial least squares
Source: BMC Bioinformatics. 2011 Aug 3;12:318. doi: 10.1186/1471-2105-12-318 (PMC3175482; doi:10.1186/1471-2105-12-318)

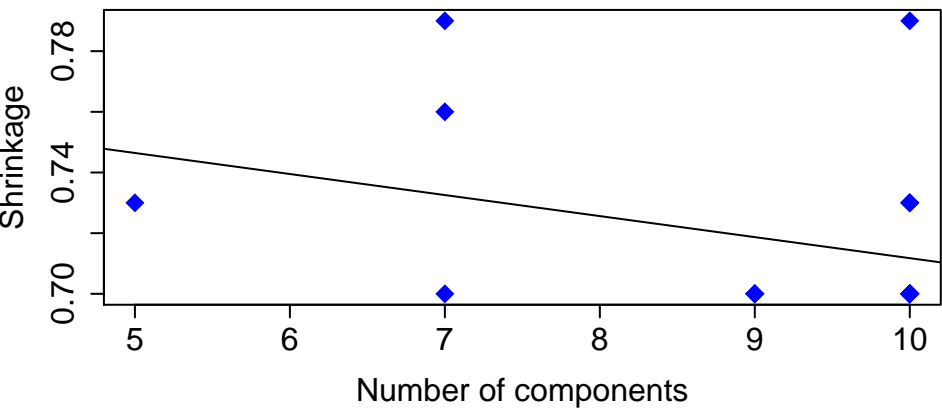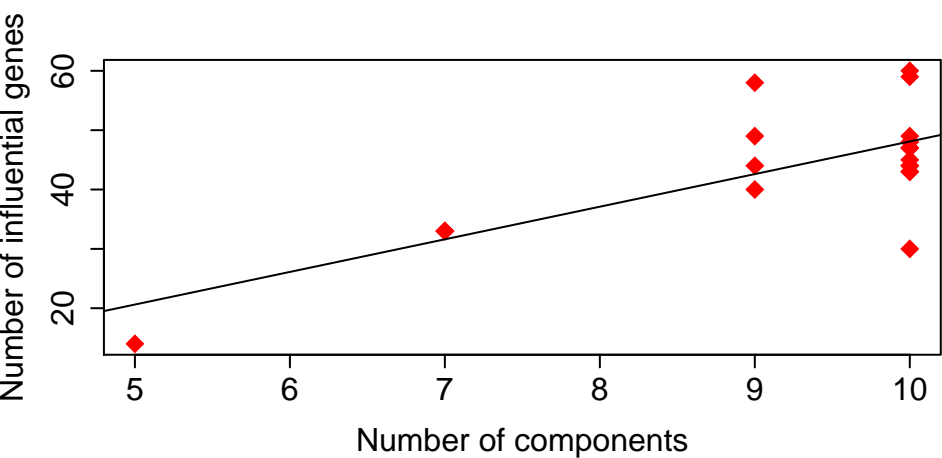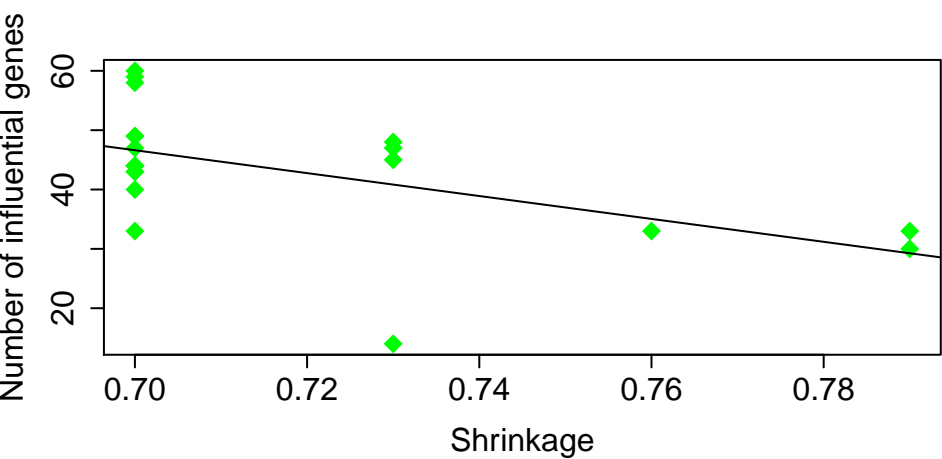

Supplement: Additional file 2 — Figure S1 - Mutual relation of shrinkage level, number of components and number of influential variables. Results obtained from the 20 ST-PLS model fits presenting the mutual relation of shrinkage level, number of components and number of influential genes. Upper panel shows the scatterplot between the shrinkage level and a number component, indicating complexity of the model increases with decrease of shrinkage level. Middle panel shows the scatterplot between number of components and number of influential genes, indicating influential genes increases with the increase of model complexity. Lower panel shows the scatterplot between shrinkage level and number of influential genes, indicating influential genes decreases with the increase of shrinkage level. [file 1471-2105-12-318-S2.PDF]
